# Azacitidine monotherapy versus combination regimens as post-HSCT maintenance therapy in high-risk myeloid malignancies: a retrospective cohort study

**DOI:** 10.1007/s12185-025-04097-8

**Published:** 2025-11-11

**Authors:** Ying Shen, Pengyu Zhang, Aili He, Jianli Wang, Jie Liu, Wanhong Zhao, Liufang Gu, Jin Wang, Bo Lei, Xueying Li, Yun Yang

**Affiliations:** 1 Department of Hematology, the Second Affiliated Hospital of Xi’an Jiaotong University, Xi’an, China; 2 Xi’an Key Laboratory of Hematological Disease, Xi’an Jiaotong University, Xi’an, China; 3 National–Local Joint Engineering Research Center of Biodiagnostics & Biotherapy, the Second Affiliated Hospital of Xi’an Jiaotong University, Xi’an, China

**Keywords:** Myeloid malignancy, High-risk, Post-HSCT, Maintenance therapy, Azacitidine

## Abstract

Azacitidine (AZA) monotherapy demonstrates efficacy for post-transplant maintenance in patients with high-risk myeloid malignancies. However, no study has directly compared combination regimens. In this retrospective cohort study, 59 patients (AML = 56, MDS = 3) received AZA-based maintenance post-allogeneic hematopoietic stem cell transplantation (allo-HSCT), as monotherapy (n = 33), AZA plus interferon-α (IFN-α) (n = 15), or AZA plus targeted agents (n = 11). At the median 31-month follow-up, the overall relapse rate was 10.2% (6/59), with comparable rates across groups (AZA: 9.1%, AZA + IFN-α: 13.3%, AZA + targeted: 9.1%) (P = 0.850). Three 3-year relapse-free survival (89.4%; 95%CI 84.7–94.1%) and overall survival (84.4%; 95%CI 78.9–89.9%) rates did not differ significantly between monotherapy and combination regimens (RFS P = 0.975; OS P = 0.770). Overall adverse event rates showed no statistical difference (P > 0.05), although febrile reactions were more common with IFN-α combination regimens (P < 0.001). These findings demonstrate that AZA monotherapy has non-inferior efficacy compared with combination regimens and has a favorable toxicity profile, establishing it as a viable backbone for maintenance therapy in MRD-negative patients. Biomarker-driven combinations warrant prospective validation.

## Introduction

Acute myeloid leukemia (AML) and myelodysplastic syndromes (MDS) are common myeloid malignancies of the hematopoietic system[1]. Allogeneic hematopoietic stem cell transplantation (allo-HSCT) is currently the only method that may cure high-risk myeloid malignancies[2]. However, relapse after transplantation remains a big challenge, which has a significant impact on patients' disease-free survival (DFS) and overall survival (OS). Therefore, it is very important to find an effective method to extend the DFS period after transplantation for patients. Hypomethylating agents are currently the most widely used treatment method in myeloid malignancies, and some study has shown that the relapse rate of hypomethylating agents (azacytidine, decitabine) maintenance therapy was about 20% [3, 4]. Moreover, with the advent of the era of targeted drugs, such as B-cell lymphoma-2 (BCL-2) inhibitor (BCL-2i), FMS-like tyrosine kinase 3 (FLT3) inhibitor (FLT3i), isocitrate dehydrogenase (IDH) inhibitor, the combination of hypomethylating agents with targeted drugs for maintenance therapy is a new trend. However, no data was presented to compare monotherapy and combination therapy of demethylating agents for maintenance therapy after transplantation in high-risk myeloid malignancies. This single-center, retrospective study compared azacytidine (AZA) monotherapy with combination regimens (AZA + IFN-α or AZA + targeted agents) in high-risk AML and MDS patients who achieved complete remission (CR) with minimal residual disease (MRD) negativity post allo-HSCT. The aim of this study was to explore the efficacy of different composite regimens based on AZA, clarify whether AZA in combination with interferon or targeted agents is superior to AZA monotherapy for post-HSCT maintenance therapy.

## Materials and methods

### Study design

This is a retrospective cohort study performed on patients with myeloid poor-risk malignant tumor (AML[5], MDS[6]) who received allo-HSCT between March 2019 and December 2022 in the Second Affiliated Hospital of Xi’an Jiaotong University (Xi’an, China). Approval for this analysis was obtained from the Ethics Committee of the Second Affiliated Hospital of Xi’an Jiaotong University (Ethics number: 2017010).

The choice of combination therapy (AZA + IFN-α or AZA + targeted agents) was based on individualized clinical decisions, including molecular profiles (e.g., FLT3-ITD status for sorafenib), prior treatment response, and physician–patient shared decision-making. Patients included in the AZA monotherapy cohort (named AZA group) received AZA at a dosage of 75 mg/m^2^ on days 1 through 5 of the 28-day treatment cycle, for a total of 12 cycles. In the AZA combination therapy cohort with interferon (IFN-α group), the treatment regimen consisted of recombinant human interferon alpha (named IFN-α) at a dosage of 300 IU (subcutaneous injection) administered every other day for two weeks, in combination with AZA at a dosage as above, with a 28-day cycle. In the AZA combination therapy cohort with targeted drugs (named targeted drugs group), the method of AZA usage was the same as previously described, with the addition of venetoclax (200 mg/day orally for 7–14 days) or FLT3i sorafenib (400 mg twice daily orally) based on the patient's condition. The treatment cycle was 28-day, for a total of 12 cycles.

### Inclusion and exclusion criteria

Inclusion criteria called for individuals with high-risk myeloid malignancies (AML, MDS), based on cytogenetic and/or molecular profile at diagnosis, underwent matched or mismatched, related or unrelated, bone marrow or peripheral blood, cord blood, or haploidentical allo-HSCT, and with no evidence of morphological disease by peripheral blood and bone marrow aspiration after transplantation. Patients with adverse risk category by cytogenetics and molecular profiles based on the European LeukemiaNet risk stratification were defined as poor-risk AML [7]. Patients with an IPSS-R score greater than 4.5 by using cytogenetics and molecular profiles based on the Revised International Prognostic Scoring System (IPSS-R) were defined as poor-risk MDS [8]. We defined negative MRD status as the absence of detectable tumor cells using flow cytometry (with a sensitivity of 10^–5^). MRD was assessed via multiparameter flow cytometry (MFC), following EuroFlow protocols.

### Statistical analysis

Relapse-free survival (RFS) was defined as the time from the initiation of maintenance therapy to relapse or death. Overall survival (OS) was defined as the period between maintenance initiation date and the date of death or last follow-up. The relapse rate is the percentage of patients who relapsed within the period of follow-up. For overall survival analysis, we censored patients still alive at the time of data analysis, and for time to relapse analysis, we censored patients who did not have a relapse at the time of data analysis. OS and RFS analysis were performed using the Kaplan–Meier method, with the log-rank test selected as the statistical method. Considering that two patients had already died before the recurrence of the disease, a competing risk analysis was done to compare the association with different therapy among causes of relapse, with Gray’s test for selected as the statistical method.

Multivariable Cox proportional hazards models were constructed to adjust for potential confounders, including time from allo-HSCT to maintenance initiation, pre-transplant MRD status, age, disease subtype (AML/MDS), and conditioning regimen. Hazard ratios (HRs) with 95% confidence intervals (CIs) were calculated to evaluate independent predictors of RFS and OS.

Student* t* test was used to determine differences in continuous variables and the chi-square test was used to determine differences in categorical covariates. All tests were two-sided, and P < 0.05 were defined as a significant difference. All statistical analyses were performed with Statistical Product and Service Solutions (SPSS) Statistics for Windows, Version 18.0 (IBM Corp., Armonk, NY, USA) and GraphPad Prism 8 (GraphPad Software, USA).

## Results

### Patients and treatment

A total of 59 individuals were included in this retrospective study, comprising 56 cases of AML and 3 cases of MDS. All MDS patients received hypomethylating agents (azacitidine or decitabine) as induction therapy prior to HSCT, with 1/3 achieving complete remission (CR) and 2/3 with hematologic improvement. The median age of the cohort was 41 years (ranging from 9 to 65 years), with a median of 4 cycles (1 to 9) of pre-transplant therapy. The median number of CD34^+^ cell in peripheral blood hematopoietic stem cells was 6.16 × 10^6^/kg (ranging from 2.13 × 10^6^/kg to 34.17 × 10^6^/kg), and the median time to neutrophil engraftment was 11 days (ranging from 10 to 25 days), while the median time to platelet engraftment was 12 days (ranging from 8 to 33 days). Maintenance therapy was initiated approximately on the 120th day post-transplant (ranging from 30 to 351 days), with the median number of maintenance therapy lines being 4 cycles. A total of 33 patients received AZA monotherapy maintenance treatment, while 26 patients received AZA-based therapy. In combination group, there were 15 cases received AZA combined with IFN-α, 8 cases with sorafenib, 3 cases with venetoclax.

The characteristics of the cohort receiving different maintenance therapy were presented in Table 1. In different maintenance subgroups, the initiation time of maintenance therapy varies among the groups. The median start time for maintenance treatment in the IFN-α group was 94 days after transplantation (30–195 days), while the times for maintenance treatment in the AZA group and the targeted drugs group were 133.5 days and 122.5 days after transplantation, respectively.

**Table 1:** Characteristics of patients receiving different maintenance therapy

Characteristics	AZA (n = 33)	AZA + IFN-α (n = 15)	AZA + targeted drugs (n = 11)	*P*
Sex, male/female	16/17	10/5	7/4	0.487
Age at allo-HSCT in years, median(range)	40 (9–65)	42.5 (11–57)	34 (16–57)	0.712
Type of disease, n (%)				1.000
AML	31 (93.9)	14 (93.3)	11 (100)	
MDS	2 (6.1)	1(6.7)	0	
Disease status at allo-HSCT, n (%)				0.287
CR1	30 (90.9)	12 (80.0)	10 (90.9)	
CR2	0	1 (6.7)	1 (9.1)	
NR	3 (9.1)	2 (13.3)	0	
Days from allo-HSCT to maintenance therapy, median (range)	133.5 (30–314)	94 (30–195)	122.5 (60–351)	0.027*
Conditioning regimen, n (%)				0.052
BU CY	24 (72.7)	11 (73.3)	6 (54.5)	
BU FLU	0	2 (13.3)	1 (9.1)	
BU CY FLU	9 (27.3)	1 (6.7)	4 (36.4)	
Other	0	1 (6.7)	0	
Donor-recipient sex matched, n (%)				0.699
Yes	20 (60.6)	11 (73.3)	8 (72.7)	
No	13 (39.4)	4 (26.7)	3 (27.3)	
Donor type, n (%)				0.286
HLA-haploidentical donor	23 (69.7)	14 (93.3)	9 (81.8)	
HLA-matched sibling donor	6 (18.2)	0	2 (18.2)	
HLA-unrelated donor	4 (12.1)	1 (6.7)	0	
Stem cells source, n (%)				0.070
PB	29 (87.9)	9 (60.0)	10 (90.9)	
PB + BM/cord blood	4 (12.1)	6 (40.0)	1 (9.1)	
Cell dose, median (range)	11.20 (5.9–16.44)	9.94 (5.88–16.44)	10.49 (7.43–15.56)	0.684
CD34^+^ cell dose (× 10^6^/kg) of PBSCT, median(range)	6.16 (2.12–34.17)	6.66 (2.13–12.91)	5.36 (3.43–10.26)	0.758
MRD before HSCT, n (%)				0.905
Negative	27 (81.8)	11 (73.3)	9 (81.8)	
Positive	6 (18.2)	4 (26.7)	2 (18.2)	
Treatment cycles before HSCT, median (range)	4 (1–9)	4 (2–8)	5 (3–7)	0.429
Time to neutrophil engraftment (days), median (range)	11 (10–15)	12 (10–25)	11 (11–14)	0.705
Time to platelet engraftment (days), median (range)	12 (8–19)	12.5 (8–33)	12.5 (9–25)	0.114

*AML* acute myeloid leukemia, *BM* bone marrow, *BU* busulfan, *CY* cyclophosphamide, *CR* complete remission, *FLU* fludarabine, *GVHD* graft-versus-host disease, *HSCT* hematopoietic stem cell transplantation, *HLA* Human leukocyte antigen, *MRD* minimal residual disease, *PB* peripheral blood, *PBSCT* peripheral blood stem cell transplantation, *NR* no reponse. * *P *< 0.05

### Efficacy

**Fig. 1:**
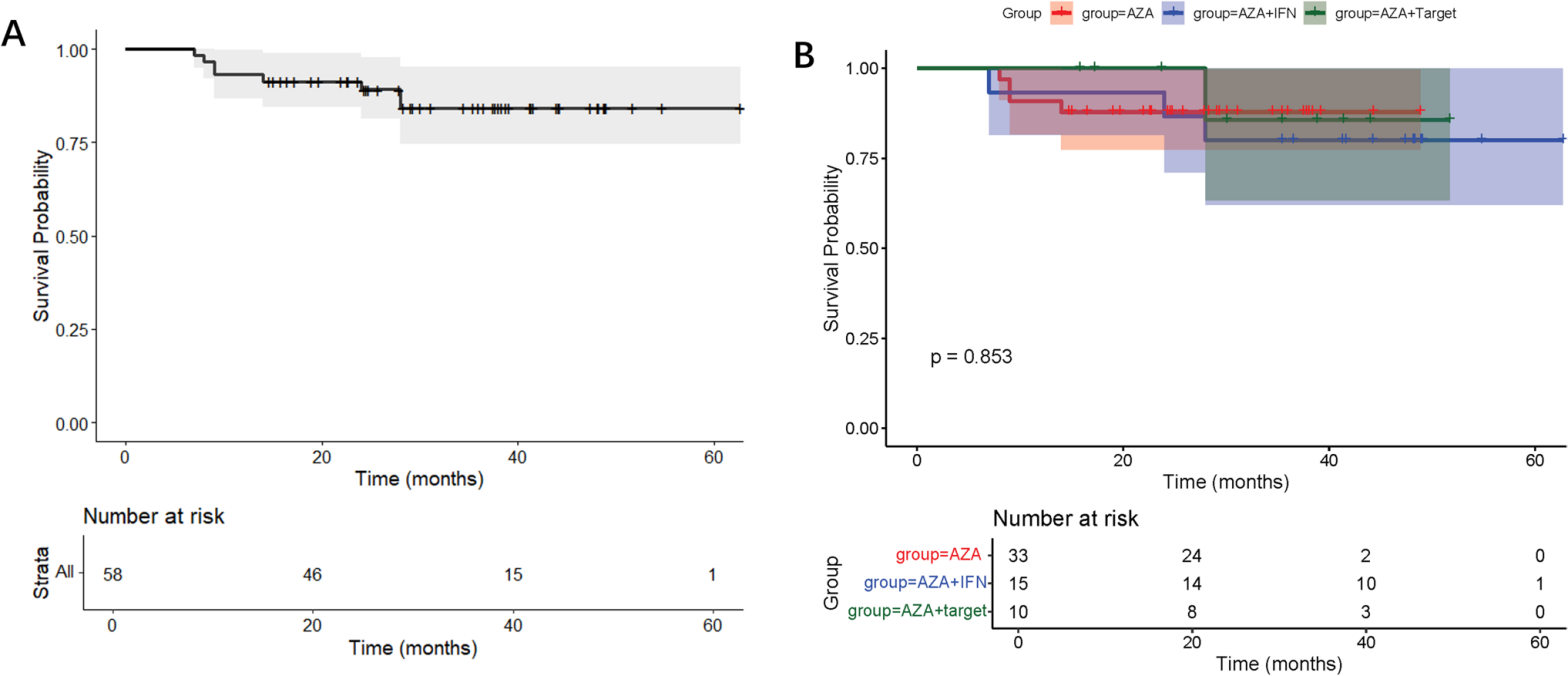
Overall survival (OS) analysis. **a** Overall cohort. **b** Subgroups (AZA monotherapy, AZA + IFN-α, AZA + targeted agents)

**Fig. 2:**
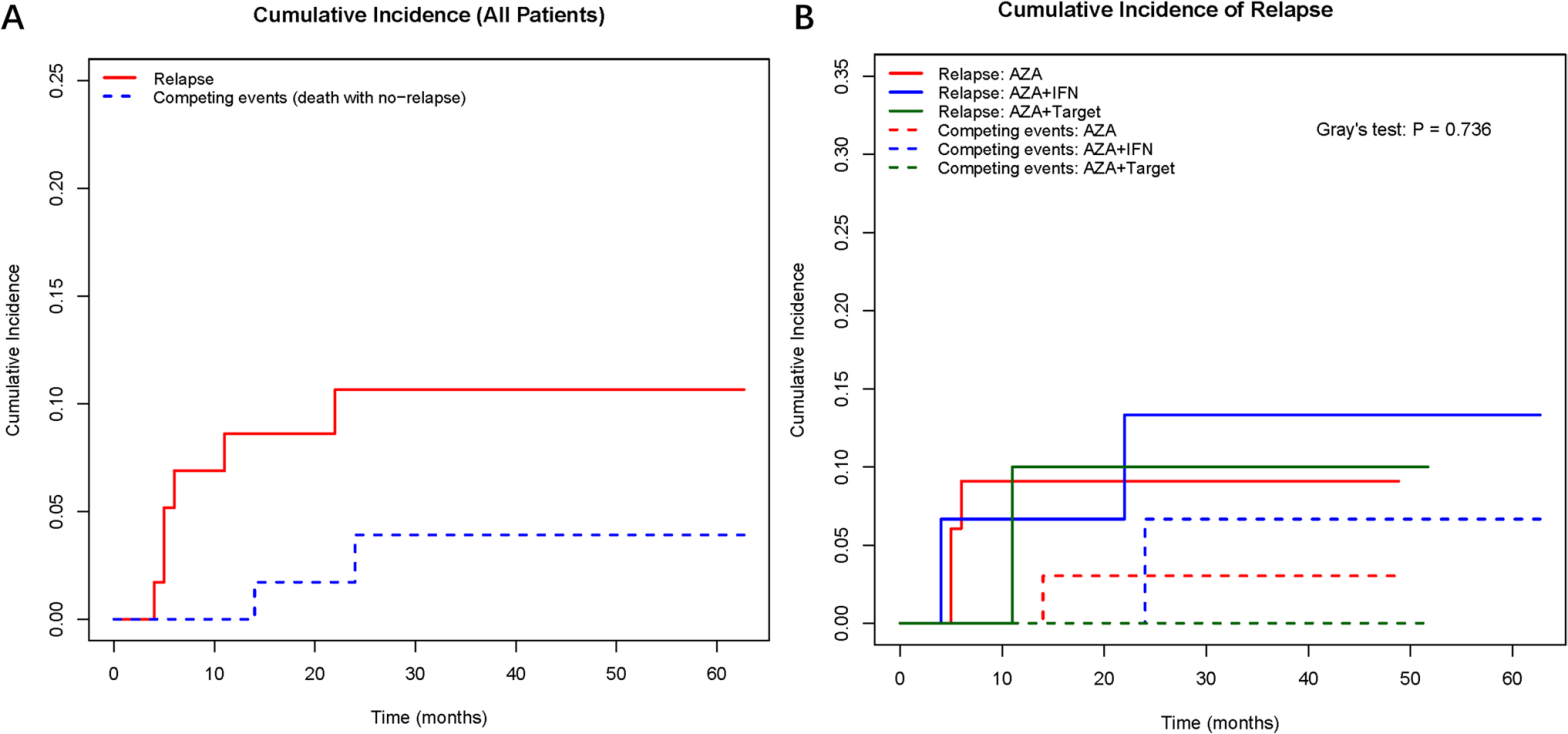
Competing risk model analysis of cumulative relapse rate. **a** Cumulative relapse rate of the entire cohort. **b** cumulative relapse rate of patients in different strata (AZA monotherapy, AZA + IFN-α, AZA + targeted agents)

The median post-maintenance therapy follow-up duration was 31 months (range 7–63). By the end of the follow-up, there were 6 cases of disease recurrence, including 5 cases of AML (5/56, 8.9%) and 1 case of MDS (1/3, 33.3%). The relapse rate in the AZA group was 9.1% (3/33), while the relapse rates for the IFN-α group and the targeted drugs group were 13.3% (2/15) and 9.1% (1/11), respectively (*P* = 0.850, Fig. 1). The median time from maintenance therapy to relapse in the overall cohort and each maintenance therapy group had not been reached, and there were no significant differences in RFS rates from maintenance therapy among the groups (*P* = 0.975). Three-year RFS were 90.9%, 86.7% and 80.0% respectively. In a competing risk analysis, the association of relapse with different therapy did not statistically differ among causes of death (*P* = 0.736, Fig. 2).

A total of 8 patients died by the end of the follow-up, including 7 cases of AML (7/56, 12.5%) and 1 case of MDS (1/3, 33.3%). The mortality rate in the AZA group was 12.1% (4/33), while the mortality rates for the IFN-α group or targeted drugs group were 20.0% (3/15) and 9.1% (1/11), respectively (*P* = 0.770). The median OS time from maintenance therapy in the overall cohort and each maintenance therapy group had not been reached (Fig. 2), and there were no significant differences in OS among the groups (*P* = 0.9237). The main causes of death were disease recurrence and progression, with one death due to severe pneumonia and another due to obliterative bronchiolitis. Three-year OS were 87.9%, 80.0% and 90.9% respectively.

### Donors and grafts

Most patients received HLA-haploidentical donor stem cell (78.0% overall; 23/33 in the AZA group, 14/15 in the IFN-α group, 9/11 in the targeted drugs group). Neither donor type (*P* = 0.286) nor stem cell dose (*P* = 0.070) differed among groups.

Univariate analysis for the effect of donor types showed no difference between study groups in terms of either OS (hazard ratio [HR] = 0.62; 95% confidence interval [CI], 0.12–3.31; *P* = 0.574) or time to relapse (HR = 0.66; 95% CI, 0.12–3.66; *P* = 0.635).

In terms of stem cell source, most patients received peripheral blood stem cells (81.4% overall; 29/33 in the AZA group, 9/15 in the IFN-α group, 10/11 in the targeted drugs group), and there was no statistical difference among the groups (*P* = 0.070); univariate analysis also showed no difference in either OS (HR = 0.91; 95% CI, 0.18–4.62; *P* = 0.912) or time to relapse (HR = 0.71; 95% CI, 0.08–6.13; *P* = 0.753).

### MRD status

Prior to transplantation, 79.7% of all patients were MRD negative (27/33 in the AZA group, 11/15 in the IFN-α group, 9/11 in the targeted drugs group); there was no difference among the study groups (*P* = 0.905). Patients with detectable pre-transplant MRD exhibited a significantly elevated risk of relapse (HR = 7.96, 95% CI: 1.45–43.61, *P* = 0.017) and a trend toward increased mortality (HR = 3.72, 95% CI: 0.93–14.94, *P* = 0.064) compared to MRD-negative patients. All patients maintained a negative MRD prior to maintenance therapy.

### Chronic graft-versus-host disease (cGVHD)

As shown in Table 2, before the initiation of maintenance therapy, a total of 33.9% of individuals had cGVHD to varying degrees (8/33 in the AZA group, 7/15 in the IFN-α group, 5/11 in the targeted drugs group). In the period of maintenance therapy, 45.8% of individuals were diagnosed with cGVHD (13/33 in the AZA group, 8/15 in the IFN-α group, 6/11 in the targeted drugs group). There was no difference in the numbers of patients diagnosed with cGVHD among the groups (P = 0.432). Inclusion of cGVHD in the cause of death analysis revealed no statistical difference among study groups (P = 0.961). There was no patient went through acute GVHD.

**Table 2:** Characteristics of cGVHD during maintenance therapy

Characteristics	AZA(n = 33)	AZA + IFN-α(n = 15)	AZA + targeted drugs (n = 11)	*P*
Severity of cGVHD, n (%)				0.443
Severe	2 (6.1)	3 (20.0)	0	
Moderate	1 (3.0)	0	0	
Mild	10 (30.3)	5 (33.3)	6 (54.6)	
None	20 (60.6)	7 (46.7)	5 (45.4)	
Number of sites, n (%)				
0	19 (57.6)	7 (46.7)	5 (45.4)	0.928
1	8 (24.2)	4 (26.7)	4 (36.4)	
2	5 (15.2)	3 (20.0)	2 (18.2)	
≥ 3	1 (3.0)	1 (6.7)	0	
Site of cGVHD, n				0.185
Skin	12 (60.0)	5 (35.7)	6 (54.6)	
Eye	0	3 (21.4)	0	
Liver	2 (10.0)	1 (7.1)	0	
Gut	6 (18.2)	3 (21.4)	2	
Lung	0	2 (14.3)	0	

*cGVHD* chronic graft-versus-host disease

### Adverse events

No unexpected adverse events (AEs) were observed across the treatment groups. Hematologic toxicities were prevalent but varied by regimen: in the AZA monotherapy group (n = 33), 66.7% experienced hematologic events, predominantly neutropenia (63.6% any grade; 36.4% grade ≥ 3) and thrombocytopenia (66.7% any grade; 27.3% grade ≥ 3) with no severe anemia. The AZA-IFN-α group (n = 15) showed higher-grade neutropenia (60.0% grade ≥ 3) and thrombocytopenia (60.0% any grade; 20.0% grade ≥ 3), while the AZA-targeted group (n = 11) had moderate neutropenia (63.6% any grade; 18.2% grade ≥ 3). Non-hematologic toxicities included transient fever (66.7% in IFN-α vs. 9.1% in targeted), frequent nausea (90.9% AZA, 73.3% IFN-α, 90.0% targeted), and respiratory infections (24.2% AZA, 20.0% IFN-α, 9.1% targeted). Hepatic involvement manifested as elevated ALT (any grade in 30.3–36.4% across groups). Crucially, no grade ≥ 3 non-hematologic events occurred, and hematologic grade ≥ 3 events were confined to neutropenia. Among the 8 patients treated with sorafenib combined with azacitidine, 2 cases had concurrent skin damage, and 1 case had secondary hypertension. Overall AE incidence showed no significant difference between groups (*P* > 0.05), though IFN-α combinations demonstrated higher febrile reactions, while targeted regimens exhibited the most favorable safety profile.

Dose reductions due to AEs occurred in 3/15 (20.0%) of IFN-α group (fever management) and 3/11 (27.3%) of targeted group (cytopenia, 2/8 in FLT3i group, 1/3 in BCL2i group). No AZA monotherapy patients required dose reduction, but 9 patients extended the treatment interval due to cytopenia (from 28 to 40 days).

## Discussion

Relapse remains the leading cause of treatment failure in high-risk myeloid malignancies undergoing allo-HSCT, occurring in around 35–45% of patients, and portending a poor prognosis [9]. Hypomethylating agents, especially AZA, have shown favorable efficacy and tolerability as induction therapy in AML patients, especially elderly patients unable to tolerate intensive chemotherapy [10]. In some prospective and retrospective study, the authors demonstrated that maintenance therapy with hypomethylating agents after allo-HSCT was tolerance and could decrease relapse rate in high-risk AML and MDS patients [11–16]. On the contrary, some studies suggested that AML patients might not benefit from maintenance therapy with hypomethylating agent post-transplantation [17, 18], may due to the short period of treatment and follow-up.

Besides hypomethylating agents, a lot of new targeted drugs, such as venetoclax (BCL-2i) [19], sorafenib (FLT3i) [20], were used in maintenance therapy after allo-HSCT in AML or MDS, with or without hypomethylating agent. One study reported that low-dose decitabine plus venetoclax was safe and effective as post-transplant maintenance therapy for high-risk AML and MDS, with 2-year OS and event-free survival (EFS) rates of 85.2% and 84.7% [19]. Sorafenib monotherapy maintenance therapy reduces the risk of relapse and death after allo-HSCT for FLT3-ITD-positive AML, with 2-year RFS probability was 85.0% [20].

IFN-α, exerting a relatively strong immunomodulatory effect, can kill AML cells by regulating T cell and natural killer cell functions [21]. IFN-α have the ability to induce a graft-versus-leukemia (GVL) effect and clear tumor cells in patients after allo-HSCT [22]. Based on this, IFN-α was used in preemptive therapy in MRD-positive patient post-transplantation, and achieved similar clinical outcomes as donor lymphocyte infusion (DLI) [23]. It is reported that the cumulative incidence of achieving MRD negative state at 2 years after IFN-a treatment was 78.2% [24]. However, there was no data about the prophylactic therapy efficacy of IFN-a.

In the present study, we first directly comparing AZA monotherapy with AZA combined with immunotherapy (IFN-α) and targeted agents in an MRD-negative cohort of high-risk myeloid malignancies after allo-HSCT. Our center employs a treatment selection driven by both biomarker profiles and clinical feasibility: 1) FLT3-ITD-positive AML patients receive AZA plus FLT3 inhibitors (sorafenib; n = 8) to target oncogene-dependent clones [20]; 2) haploidentical transplant recipients without active GVHD receive AZA plus IFN-α (n = 15) to augment graft-versus-leukemia effects with minimal GVHD risk [24]; 3) for venetoclax combinations (n = 3), selection requires adequate hematologic recovery (ANC ≥ 1.0 × 10⁹/L and platelets ≥ 50 × 10⁹/L) as well as patient affordability, given its empirical use for broad antileukemic activity [25]; 4) all others receive AZA monotherapy (n = 33) as the efficacy-balanced backbone.

We observed a 3-year RFS and OS after AZA-based maintenance therapy of 89.4% and 84.4%, respectively. The favorable outcomes observed in our cohort may be attributed to stringent MRD-negative inclusion criteria and early initiation of maintenance therapy. Importantly, no statistically significant differences in OS or RFS were observed between the AZA monotherapy and combination regimen groups. However, it is critical to emphasize that due to the limited sample size and low number of events, the study was underpowered to detect anything but very large differences in survival. Therefore, these non-significant results cannot be interpreted as evidence of equivalence, nor do they rule out the possibility of clinically meaningful differences among the regimens.

Compared to a prospective Asian trial using lower-dose AZA (20–50 mg/m^2^ ×  5 d) [26], our intensified regimen (75 mg/m^2^ ×  5 d) demonstrated superior survival outcomes: 3-year OS of 87.9% versus 2-year OS of 77.0%, and 3-year PFS of 90.9% versus 2-year PFS of 73.3%. Notably, this survival advantage was achieved without significantly increasing severe hematologic toxicity, as evidenced by comparable rates of grade 3–4 cytopenia (36.4% in our cohort vs. 33.3% in the trial). These findings suggest that moderate dose escalation of AZA maintenance therapy (75 mg/m^2^) may enhance long-term survival while maintaining an acceptable safety profile in high-risk post-transplant patients (Table 3).

**Table 3:** Adverse events during maintenance therapy

Event	AZA group (n = 33)		AZA-IFN-α group (n = 15)		AZA-targeted drugs group (n = 11)		P value	
	Any grade	Grade ≥ 3	Any grade	Grade ≥ 3	Any grade	Grade ≥ 3	Any grade	Grade ≥ 3
Neutropenia	21(63.6)	12 (36.4)	11 (73.3)	9 (60.0)	7 (63.6)	2 (18.2)	0.865	0.087
Anemia	18 (54.5)	0	9 (60.0)	0	8 (72.7)	0	0.631	
Thrombocytopenia	22 (66.7)	9 (27.3)	9 (60.0)	3 (20.0)	7 (63.6)	0	0.932	0.148
Fever	3 (9.1)	0	10 (66.7)	0	1 (9.1)	0	< 0.001*	
Nausea	30 (90.9)	0	11 (73.3)	0	10 (90.9)	0	0.256	
Vomiting	9 (27.3)	0	8 (53.3)	0	5 (45.5)	0	0.188	
Diarrhea	2 (6.1)	0	4 (26.7)	0	1 (9.1)	0	0.120	
Increased ALT level	10 (30.3)	2 (6.1)	5 (33.3)	2 (13.3)	4 (36.4)	1 (9.1)	0.931	0.698
Increased blood Cr level	0	0	1 (6.7)	0	0	0	0.441	
Electrolyte disorders	11 (33.3)	0	4 (26.7)	0	5 (45.5)	0	0.607	
Respiratory system infection	8 (24.2)	0	3 (20.0)	0	1 (9.1)	0	0.618	

*ALT* glutamic pyruvic transaminase, *Cr* creatinine. * *P* < 0.05

MRD assessment in AML has an established role in disease prognostication [27]. Serial MRD assessment is now routinely recommended in the evaluation of treatment response and monitoring in AML by the European LeukemiaNet [28]. Our cohort includes 47 patients with negative MRD and 12 with positive MRD before transplantation, but all the patients achieved MRD negativity before maintenance initiation. In MORPHO Study which evaluated the efficacy and safety of maintenance therapy with gilteritinib after allogeneic SCT showed that gilteritinib was significantly superior to placebo only in patients with positive MRD before transplantation[29]. In our study, we used sorafenib as the drug for the maintenance therapy regimen, not gilteritinib. The evidence for the use of sorafenib are as follows. In SORMAIN study, regardless of the MRD status on day 30 after transplantation (negative or positive), patients receiving sorafenib maintenance had significantly better RFS than those receiving placebo [20]. In another open-label, multicenter, randomized, phase 3 trial, the same result was showed. Among patients who were MRD-negative at both day 30 and day 90 after transplantation, the sorafenib group showed significantly better progression-free survival (PFS) and OS compared with the placebo group. Regardless of the early post-transplant MRD status (negative or positive), patients receiving sorafenib maintenance had significantly better PFS and OS than those receiving placebo [30]. As for azacitidine (AZA), the RELAZA2 trial showed that AZA preemptive therapy prevented hematologic relapse in 58% of high-risk MDS/AML patients who developed MRD after HSCT [15]. Our approach initiates therapy before MRD emergence, potentially extending this protective effect. Furthermore, we found that participants with detectable MRD had a 7.96 times greater hazard of relapse and 3.72 times greater hazard of death than MRD-negative patients before transplantation. Therefore, in pre-transplant MRD-positive patients, it is essential to start maintenance therapy as early as possible after transplantation.

Cytopenia was the most significant toxicity during maintenance initiation, with grade 3–4 events occurring in 18.2–60.0% of patients across regimens. This hematologic suppression increased infection susceptibility, evidenced by respiratory infections in 9.1–24.2% of cases. Non-hematologic toxicities, such as primarily fever, nausea and transaminitis, were generally manageable. Only fever incidence was more common in AZA plus IFN-α group, while other toxicities showed comparable rates. Within the AZA-sorafenib subgroup, 25% (2/8) patients required dose reductions (due to cytopenias [n = 1] and rash [n = 1]), with no treatment discontinuations occurred. Our dose reduction rate was lower than landmark trials SORMAIN (48% with sorafenib) [20] and MORPHO (32% with gilteritinib) [29], potentially due to proactive supportive care.

There were several limitations to our study. First, there was an absence of a direct comparative cohort without maintenance therapy in our center. The most important reason was historical heterogeneity in our center's early transplant practices (pre-2019), where maintenance strategies were inconsistently applied based on targeted drug availability and GVHD status, while contemporary protocols universally incorporate azacitidine for high-risk patients. This fundamental shift in standard-of-care, compounded by substantial evolution in conditioning regimens, introduces irreconcilable confounding factors that preclude meaningful statistical adjustment. Second, the modest cohort size constrained subgroup analyses: the MDS population was limited to 3 patients, and targeted therapy subgroups (venetoclax n = 3; sorafenib n = 8) were underpowered to detect regimen-specific differences. So, we pooled venetoclax and sorafenib as targeted group, which might mask agent-specific effects and obscure unique toxicity or efficacy profiles. The single relapse in the AZA plus venetoclax subgroup (1/3) warrants caution in interpreting this regimen's efficacy, though underpowered for formal analysis. Future multi-center studies with larger cohorts are needed to delineate optimal drug-specific combinations. Third, the median follow-up of 31 months may underestimate late relapses in diseases like MDS with indolent progression patterns.

In summary, this study established that AZA-based maintenance therapy—whether as monotherapy or combined with immunotherapy/targeted agents—achieves exceptional 3-year survival in rigorously selected MRD-negative high-risk myeloid malignancy patients post-allo-HSCT. While limited by retrospective design and subgroup sizes, these findings provide a clinically actionable framework for personalizing post-transplant maintenance and clues for subsequent clinical trials on post-transplant maintenance regimen selection.

## Data Availability

The data that support the findings of this study are available on reasonable request from the first author.
